# Floquet engineering of Feshbach resonances in ultracold gases

**DOI:** 10.1126/sciadv.adw3856

**Published:** 2025-10-03

**Authors:** Alexander Guthmann, Felix Lang, Louisa Marie Kienesberger, Sian Barbosa, Artur Widera

**Affiliations:** Department of Physics and Research Center OPTIMAS, RPTU Kaiserslautern-Landau, 67663 Kaiserslautern, Germany.

## Abstract

Scattering resonances are fundamental to many areas of physics, occurring across a wide range of energy scales. In ultracold quantum gases, magnetic Feshbach resonances have transformed quantum many-body research by enabling precise interaction control between atoms. Here, we demonstrate unprecedented control to engineer Feshbach resonances at tunable positions via Floquet driving in a lithium-6 (^{6}Li) atom gas, achieved through strong magnetic field modulation at megacycles per second frequencies. This periodic modulation creates scattering resonances whenever dressed molecular levels cross the atomic threshold. By adding a second modulation at twice the base frequency, we tune the asymmetry of resonance loss profiles and suppress two-body losses from Floquet heating. This technique enhances control over atomic interactions, expanding possibilities for quantum simulations of complex systems and studies of exotic quantum phases.

## Teaser

Floquet engineering of Feshbach resonances enables the precise control of atomic interactions in ultracold quantum gases.

## INTRODUCTION

When a quantum mechanical particle scatters from a potential, for certain incident energies, the scattering cross section, summarizing the effect of the scattering event, shows a divergence. These scattering resonances have profound consequences and are a ubiquitous phenomenon in nature, occurring across a broad range of energy scales and systems, including the Hoyle state in nuclear fusion within stars (*1*, *2*), the production of the Higgs boson (*3*, *4*), Kondo resonances in solid-state systems (*5*), enhanced reaction rates in molecular collisions (*6*–*8*), and low-energy quantum systems such as ultracold atomic gases.

At low collision energies, the microscopic interactions are governed by the *s*-wave scattering length $a_{s}$ (*9*, *10*). For purely elastic scattering, $a_{s}$ is real, but in the presence of inelastic events, it becomes complex with an imaginary component (*11*). The observation of magnetically tunable Feshbach resonances in a cold atom system (*12*) sparked an explosion of research applications for ultracold quantum many-body systems, as it allows to change $a_{s}$ in both strength and sign through the external magnetic field.

**Fig. 1.**
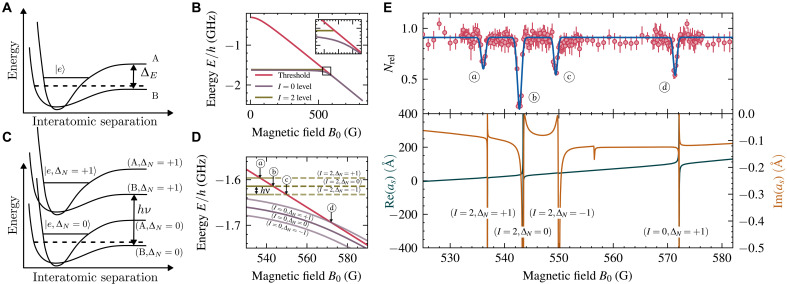
Occurence of Floquet-Feshbach resonances. (**A**) Simplified sketch of the two-channel model for Feshbach resonances. An atom pair collides on potential B, coupled to potential A with a bound state ∣e〉. A Feshbach resonance occurs when the colliding pair is resonant with ∣e〉. If the potentials correspond to molecular configurations with different magnetic moments, then their energy shift $\Delta_{E}$ can be tuned via the magnetic bias field. (**B**) Magnetic field dependence of the two highest molecular levels and the atomic threshold of two ^6^Li atoms in the lowest hyperfine states ∣a〉 and ∣b〉. The two accessible *s*-wave Feshbach resonances correspond to molecular levels with total nuclear spin I=2 [narrow resonance at 543.28 G (0.054328 T)] (*72*) and I=0 [broad resonance at 832.18 G (0.083218 T)] (*76*). At low collision energies, a Feshbach resonance occurs when a molecular state crosses the atomic threshold. Inset highlights the I=2 resonance. (**C** and **D**) Modulating the magnetic field dresses the molecular levels, and a Floquet-Feshbach scattering resonance occurs when a dressed molecular state crosses the atomic threshold. Arrows indicate resonance positions, with dashed lines indicating the continuation of the I=2 scattering pole into the continuum. (**E**) Floquet-Feshbach resonances at 18.15 MHz modulation with $B_{\text{rf}}^{\left(a\right)}\approx 3$ G. The top panel shows experimental loss spectroscopy data, fitted with Gaussians to extract resonance positions. The bottom panel displays the calculated real (blue) and imaginary (ochre) parts of the *s*-wave scattering length $a_{s}$, both showing resonant features corresponding to atom loss. Error bars represent SD over at least five measurements per data point.

This is accomplished by bringing a bound state of one molecular ground-state potential into resonance with another hyperfine molecular potential as schematically shown in Fig. 1A. One of the most notable applications of this tunability is in exploring the Bose-Einstein condensate (BEC)–Bardeen-Cooper-Schrieffer (BCS) crossover, where Feshbach resonances enable a smooth transition between a BEC of tightly bound molecules and a BCS superfluid of loosely bound Cooper pairs (*13*–*15*).

The position of magnetic Feshbach resonances is determined by the magnetic field value where the energy of the bound state crosses the atomic threshold, as shown in Fig. 1B for ^6^Li, and the width and position are dictated by the properties of the interaction potentials of the molecular dimer together with the atomic properties of its constituents. To date, magnetic Feshbach resonances have been observed for cold atomic (*9*, *12*, *16*–*19*), ionic (*20*), and molecular (*21*, *22*) systems. However, the dependence on magnetic fields to tune the interaction strength imposes limitations, especially for atomic species with weak or inaccessible resonances.

Furthermore, the relative position of Feshbach resonances in multicomponent systems cannot be adjusted with a static magnetic field. To realize applications such as simulating color superfluidity (*23*) or (Bose-)Kondo physics in quantum gases (*24*), further control over resonance positions is crucial for adjusting intra- and interspecies interactions in multicomponent systems.

Advanced techniques extending the control of magnetic Feshbach resonances so far couple the open channel to other bound states by optical, microwave, or radio-frequency (rf) transitions, where the AC Stark shift (for optical transitions) or the AC Zeeman shift (for rf transitions) induced by the applied radiation field allows for a limited tuning of the resonance position (*25*, *26*).

Optical Feshbach resonances (*27*–*29*), where laser light is used to couple atomic states, allow for interaction control at the expense of rapid atom loss due to the short lifetimes of the excited state. While such losses can be reduced using bound-bound transitions, this method is restricted to suitable molecular transitions (*25*). Moreover, scattering parameters can be modified by coupling several bound states through rf radiation (*30*, *31*), leading to shifts of existing resonances. In addition, modulated magnetic fields have been used for rf-assisted photoassociation of molecules from the scattering continuum (*32*–*36*).

In this work, we extend this concept by coupling the scattering continuum to bound states in a controlled, tunable manner, enabling the formation of scattering resonances with substantial control over the elastic component of the scattering length $a_{s}$.

This method has been proposed and theoretically studied using rf radiation polarized perpendicular (*26*, *37*–*40*) or by modulating the magnetic field parallel to the quantization axis (*41*). In the former case, the continuum is coupled to bound states in channels with different angular momentum $M_{F}$, whereas in the latter, it is coupled to states within channels that share the same $M_{F}$, including the entrance channel itself.

Recent theoretical work has further explored the origins and properties of such Floquet scattering resonances (*42*). While offering precise control over resonance properties, experimental implementation of Floquet scattering resonances has remained elusive due to the challenge of generating sufficiently large field amplitudes, which are so far only realized in the near-field regime on atomic chips to drive fast Rabi oscillations (*43*). In the case considered here, the rf modulation effectively dresses the atomic and molecular states in a Floquet-type manner, as shown in Fig. 1 (C and D), leading to the appearance of scattering resonances at magnetic field values whenever a dressed molecular level intersects the atomic threshold. Floquet engineering, successfully applied to tailoring band structures for cold atoms in optical lattices, has demonstrated powerful control over quantum states through periodic driving (*44*–*48*). Here, we present experimental realization and tuning of such Floquet scattering resonances in a cold ^6^Li gas through strong modulation of the magnetic field parallel to the quantization axis.

Here, we present the experimental realization, theoretical modeling with coupled-channel calculations, and tuning of Floquet scattering resonances in a cold ^6^Li gas through strong modulation of the magnetic field parallel to the quantization axis. By achieving modulation strengths comparable to the modulation frequency, i.e., $\mu_{B}B_{\text{rf}}∼h\nu$, we observe the formation of higher-order resonances.

Notably, interference between different Floquet orders induces asymmetry in the loss profile of Floquet-Feshbach resonances. Furthermore, we show that this asymmetry can be tuned by introducing a two-color driving scheme, where an additional driving term at twice the fundamental frequency allows us to suppress inelastic two-body losses associated with Floquet heating. By optimizing this two-color driving scheme, we achieve minimal losses, enabling the system to exhibit hydrodynamic behavior. Conceptually, this two-color periodic modulation of the magnetic field corresponds to the first two terms of a Fourier series, with $B_{\text{rf}}^{\left(a\right)}$ and $B_{\text{rf}}^{\left(b\right)}$ defining the strengths of the first and second harmonics, respectively; further details are provided in Materials and Methods.

## RESULTS

### Tuning of Floquet-Feshbach resonances

To experimentally study Floquet-Feshbach resonances, we prepare a two-component ^6^Li gas of an incoherent, equal mixture of the two lowest hyperfine states, at a temperature of *T* < 1.0 μK. On average, the number of atoms per spin state in the optical dipole trap is $2.3\times 10^{4}$. We perform atomic loss spectroscopy by applying the Floquet drive and vary the magnetic bias field $B_{0}$. A resonance is detected by a drop in atom number, and its position is extracted through a fit. For the initial observation, an additional slow modulation (600 Hz) of the magnetic field is added to artificially broaden the scattering resonances. A typical loss spectrum obtained this way is shown in the top panel of Fig. 1E.

Several loss features can be seen, which correspond to the position of resonances in the *s*-wave scattering length $a_{s}$, depicted in the bottom panel. Theoretical predictions for both the real and imaginary parts of $a_{s}$ were obtained from time-independent coupled-channel calculations based on Floquet theory, as detailed in Materials and Methods. In addition to resonance features in the real part of the scattering length $a_{s}$, the imaginary part also shows an extremum at a resonance, leading to enhanced two-body losses at resonance, which, in turn, suppress the divergence of the real part of the scattering length (*11*).

The microscopic process for such two-body losses is the absorption of drive quanta, a mechanism commonly referred to as Floquet heating (*47*), which imparts sufficient energy for the two colliding atoms to escape the trap. A description of the underlying theoretical model is provided in Materials and Methods.

We label the resonances by the underlying dressed molecular state causing it, together with the relative number of drive quanta (Floquet order), e.g., $\left(I=0,\Delta_{N}=+1\right)$ labels the resonance caused by the I=0 level dressed by $\Delta_{N}=+1$ drive quanta.

**Fig. 2.**
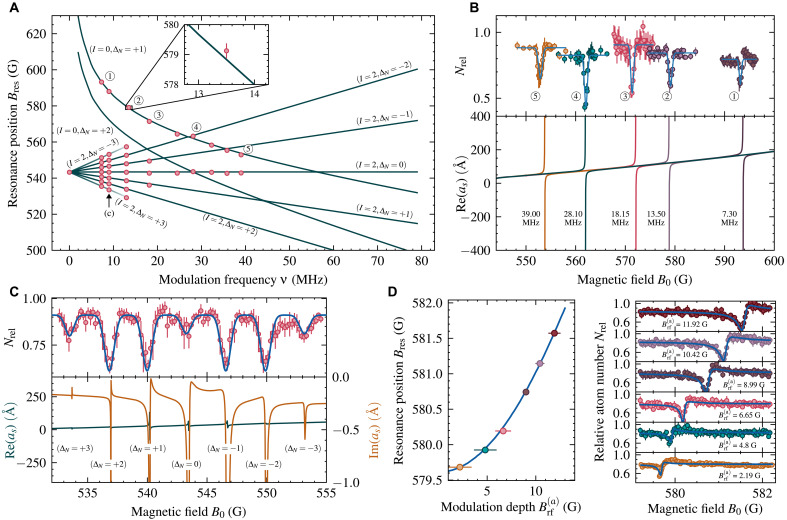
Tuning of Floquet-Feshbach resonances. (**A**) Floquet-Feshbach resonance positions as a function of modulation frequency. Solid lines show resonance position obtained from numerical coupled-channel calculations. Data points show measured resonance positions using loss spectroscopy. The error bars indicate 1σ statistical error arising due to bias coil current fluctuations, with frequency uncertainty being negligible. (**B**) Magnetic field shift of the $\left(I=0,\Delta_{N}=+1\right)$ Floquet-Feshbach resonance for five modulation frequencies. The top panel shows experimental data fitted with Gaussians, while the bottom panel shows theoretical scattering length calculations. This resonance shows the largest shift of 40.3 G between 7.3 and 39.0 MHz. (**C**) Loss spectrum and scattering length recorded at a modulation frequency of 9 MHz and a modulation strength of $B_{\text{rf}}^{\left(a\right)}\approx 5$ G showing Floquet-Feshbach resonances of the I=2 level up to order ±3. Error bars indicate 1σ of observed variation in atom number $N_{\text{rel}}$. (**D**) Dependence of the $\left(I=0,\Delta_{N}=+1\right)$ resonance’s position on the modulation strength for a driving frequency of 12.95 MHz. The left panel shows calculated (solid line) and measured resonance positions. The right panel shows atomic loss spectroscopy data taken without artificial broadening of the resonance, exhibiting a strong asymmetry. The atomic loss is fitted with a Fano profile.

The magnetic position of Floquet-Feshbach resonances depends on the driving frequency ν, as shown in Fig. 2A. Two sets of resonances for ^6^Li behave differently based on the underlying molecular state: I=2 resonances depend linearly on frequency, as the dressed I=2 states are field independent, while the atomic threshold shifts linearly in the Paschen-Back regime. This allows us to observe a ladder of Floquet resonances for small driving frequencies up to order $\Delta_{N}=\pm 3$ as shown in Fig. 2C. Interference of different orders of the Floquet drive also modifies the static resonance, which is attenuated in Fig. 2C and can even be extinguished. In contrast, the Floquet resonances derived from the I=0 resonance exhibit a pronounced nonlinear frequency dependence in Fig. 2A, shifting the resonance by more than 250 G. This behavior is due to the strong magnetic field dependence of the I=0 molecular level, as shown in Fig. 1 (B and D).

The resonance position depends not only on the driving frequency but also on the driving strength. Figure 2D shows the positional shift of the $\left(I=0,\Delta_{N}=+1\right)$ resonance with respect to the modulation strength. This shift is equivalent to an AC Zeeman effect and follows the relation $\Delta B_{\text{res}}\propto B_{\text{rf}}^{2}$ (*37*). Unlike the AC Stark shift from an oscillating electric field, this is due to an oscillating magnetic field, with increasing modulation strength shifting the resonance to higher magnetic fields. The loss data of Fig. 2D was taken without additionally broadening the resonance and therefore resolves the line shape. A clear asymmetry in the loss profile can be seen well captured by a Fano profile, first observed in the context of photoionization spectra (*49*, *50*) and later found to be applicable to many phenomena, including ultracold collisions (*51*, *52*).

### Two-color drive

Although the imaginary part of the scattering length $a_{s}$, associated with inelastic processes, helps to observe Floquet-Feshbach resonances via enhanced atom loss, these same losses also limit the usefulness of such resonances for applications. They occur because two colliding atoms have an increased likelihood of absorbing drive quanta at resonance.

The loss spectra in Fig. 1E show that the imaginary part is highly asymmetric, with distinct maxima and minima at specific magnetic fields. At the minima, the inelastic part of the scattering length becomes much smaller than the elastic part. In the present case, the asymmetry arises from interfering pathways in the Floquet-Hilbert space, where the magnetic field alters the phase between paths, resulting in constructive or destructive interference, resulting in the observed Fano profile.

**Fig. 3.**
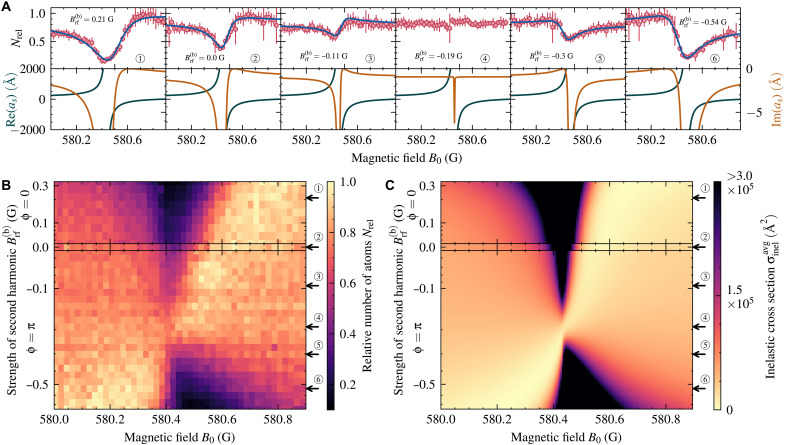
Two-color engineering of Floquet-Feshbach resonances. Effect of the additional two-color drive on the losses at the $\left(I=0,\Delta_{N}=+1\right)$ Floquet-Feshbach resonance. The strength of the first harmonic $B_{\text{rf}}^{\left(a\right)}$ and the frequency ν are fixed at 8.9 G and 13.1 MHz, respectively, while the strengths of the second harmonic $B_{\text{rf}}^{\left(b\right)}$ are varied. (**A**) Atomic loss data and theoretical scattering length for six different strengths of $B_{\text{rf}}^{\left(b\right)}$. The imaginary part of the scattering length is markedly affected by the two-color drive, while the real part is affected only very slightly. The effect of this can be seen in the reduced atomic loss. (**B**) Loss data for 36 different values of $B_{\text{rf}}^{\left(b\right)}$. The top part shows the loss spectra for increasing $B_{\text{rf}}^{\left(b\right)}$ with ϕ=0. The bottom part shows respectively the loss spectra for ϕ=π, corresponding to negative $B_{\text{rf}}^{\left(b\right)}$. (**C**) Thermally averaged inelastic scattering cross section $\sigma_{\text{inel}}^{\text{avg}}$ calculated using coupled-channel calculations, for the same range as in (B).

This interference suggests that adding more controllable pathways could further modify the asymmetry. We found that by introducing a second driving term at twice the fundamental frequency, often referred to as two-color or two-tone drive, with variable strength $B_{\text{rf}}^{\left(b\right)}$ and phase ϕ, substantial changes to the imaginary part of $a_{s}$ with minimal impact on the real part, as shown in Fig. 3, can be realized. Similar techniques have been studied for driven Hubbard systems (*53*–*56*).

For ϕ=0, increasing the amplitude $B_{\text{rf}}^{\left(b\right)}$ shifts the loss minimum slightly toward higher magnetic fields on the attractive (BCS) side of the resonance but increases losses on the repulsive (BEC) side. A more substantial effect occurs with a phase shift of ϕ=π, corresponding to a sign inversion of $B_{\text{rf}}^{\left(b\right)}$. Here, the interference causes the loss minimum to shift across the entire resonance width, moving from the attractive to the repulsive side as $|B_{\text{rf}}^{\left(b\right)}|$ increases. At an intermediate value, the loss maximum nearly vanishes, leaving only a narrow symmetric feature at the resonance center. This control over the asymmetry and position of the loss minimum allows for effective suppression of inelastic losses across both sides of the resonance, enhancing stability for applications requiring precise interaction dynamics.

### Elastic interactions

**Fig. 4.**
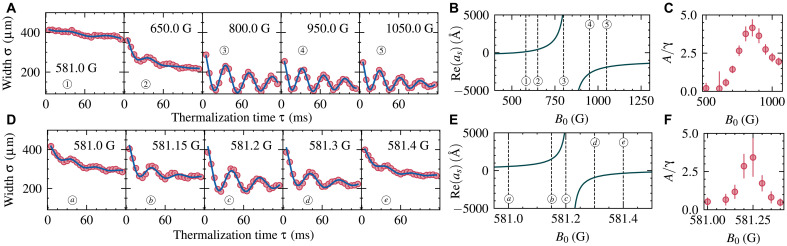
Induced elastic interactions along a Floquet-Feshbach resonance. (**A**) Evolution of the atomic cloud’s width after a sudden quench of the trap depth for various magnetic fields across the static I=0 resonance in (**D**) across the $\left(I=0,\Delta_{N}=+1\right)$ Floquet-Feshbach resonance. The magnetic field values correspond to elastic scattering lengths as indicated in (**B**) and (**E**). By fitting the cloud’s oscillatory response, we can extract oscillation amplitude A and decay rate $\gamma_{A}$. Plotting the ratio $A/\gamma_{A}$ over the magnetic field value shows typical resonance behavior, seen in (**C**) and (**F**). Error bars give fit uncertainties.

Building on this enhanced control over losses, we next confirmed the enhancement of elastic interactions at a Floquet-Feshbach resonance, which becomes more prominent as inelastic losses are suppressed. To explore this effect, we tracked cloud dynamics following a quench of the optical dipole trap depth. We use the ensuing oscillatory dynamical response as a measure for elastic interactions. Similar techniques have been used to explore collective oscillations in the BEC-BCS crossover (*57*, *58*). After preparing a cold gas, we simultaneously switch on the rf and quench the optical dipole trap to 20% of its initial depth. The case of the static I=0 Feshbach resonance serves as a reference, and we extract the dynamics of the cloud width along the axial direction of the optical dipole trap to reveal the impact of interactions. This includes not only thermalization, as the trap depth is reduced, but also the excitation of low-lying collective excitations driven by elastic interactions. Far from resonance, where interactions are weak, the cloud shows neither thermalization nor periodic dynamics. However, close to resonance in both the BEC and BCS regimes, we observe pronounced oscillations in cloud width (Fig. 4A), indicative of collective hydrodynamic behavior resulting from elastic interactions.

To induce elastic collisions at 581 G, where no such interactions are observed in the static case, we tune the modulation frequency to shift the Floquet-Feshbach resonance to this field. We induce elastic interactions at different magnetic fields around the Floquet-Feshbach resonance position while simultaneously minimizing the inelastic losses by the two-color scheme for precisely this magnetic field value. Thereby, we can induce similar oscillations at magnetic field values where the cloud previously showed no elastic response (Fig. 4, D to F). Comparing the oscillatory response between the static I=0 resonance and the Floquet-Feshbach resonance with two-color driving, we found similar dynamics, confirming that elastic scattering is enhanced under Floquet modulation, while the Floquet-induced loss and heating can be reduced to a level where still weakly damped trap dynamics can be observed.

## DISCUSSION

An exciting direction for future research is the investigation of resonance interference effects. When two Feshbach resonances approach each other, they exhibit an avoided crossing caused by coherent coupling. It has been theoretically predicted that if a control parameter exists to tune the position of Feshbach resonances, then their interference could give rise to a bound state in the continuum (BIC), a nondecaying state embedded within the scattering continuum (*59*). Floquet-Feshbach resonances, with their high degree of tunability via modulation frequency and amplitude, provide precisely such a control knob, opening a promising route to realize and explore BICs in cold atom systems.

In addition, the rapid switching of the Floquet drive offers a promising tool for exploring quenched interactions and nonequilibrium dynamics in quantum gases. We also plan to study three-body losses and to create a degenerate quantum gas on both sides of a resonance, paving the way for deeper insights into many-body physics and phase transitions in Floquet-driven quantum systems.

## MATERIALS AND METHODS

### Experimental sequence

We begin by preparing a cold gas of ^6^Li in a mixture of the two lowest hyperfine states, $|a\rangle =|f=1/2,m_{f}=+1/2\rangle$ and $|b\rangle =|f=1/2,m_{f}=-1/2\rangle$, in a steel vacuum chamber (*60*). The gas is then transported via a dipole trap with tunable focal length into a glass cell to achieve the required modulation strength for studying Floquet-Feshbach resonances. After transport, a perpendicularly polarized rf pulse is applied to equalize the mixture of states ∣a〉 and ∣b〉, followed by evaporative cooling at a bias magnetic field of 763 G to further lower the temperature and increase the density. This process results in approximately $2.3\times 10^{4}$ atoms per spin state and a density of $n_{0}=4\times 10^{14}m^{-3}$ inside the optical dipole trap. At this stage, the axial and radial trap frequencies of the cigar-shaped optical dipole trap are $\omega_{z}=2\pi \times 68$ Hz and $\omega_{r}=2\pi \times 14.3$ kHz, respectively. The temperature is chosen to be low enough to be firmly in the *s*-wave–dominated regime and to limit resonance broadening due to a thermal collision energy distribution. We do not evaporate further to ultracold temperatures because this would lead to molecule formation because of the underlying I=0 molecular state. We detect Floquet-Feshbach resonances using atomic loss spectroscopy. The magnetic field is rapidly ramped to the target bias field $B_{0}$, held for 200 ms to stabilize, and then subjected to rf modulation. For the loss spectra shown in Figs. 1E and 2 (A to C), the bias field is additionally modulated at 600 Hz with ≈ 0.7 G in amplitude, broadening the resonances for easier detection. The duration of the modulation is adjusted on the basis of its strength to optimize the signal-to-noise ratio; Table 1 lists the times used to obtain the data of Fig. 2D.

**Table 1.** Floquet modulation parameters for loss spectroscopic data. As the modulation strength increases, the losses become stronger, and hence, shorter modulation lengths are needed (Fig. 2D).

Modulation strength $B_{\text{rf}}^{\left(a\right)}$ (G)	Modulation length (ms)
2.19	1500
4.80	300
6.65	200
8.99	100
10.42	60
11.92	40

After modulation, the magnetic field is ramped back to 763 G, and the number of remaining atoms is measured via absorption imaging. We calibrate the magnetic bias field $B_{0}$ using rf spectroscopy, driving transitions between state ∣b〉 and $|c\rangle =|f=3/2,m_{f}=-3/2\rangle$. The loss spectroscopy measurement cycles are interleaved with cycles performing rf spectroscopy to account for magnetic field drifts.

For the thermalization measurements, the gas was prepared in the same way as described. After the magnetic field has been allowed to settle at the target value, the trap was instantaneously (<1 μs) quenched from 1 to 200 mW, and at the same time, for measurements on the Floquet-Feshbach resonances, the two-color drive was switched on. The amplitude of the second harmonic has been adjusted for minimal losses. The gas is held for a certain time τ with or without the Floquet drive applied, after which the gas is immediately imaged using absorption imaging at the current magnetic field.

### Scattering Hamiltonian

The total Hamiltonian H(t) of two scattered atoms in the center-of-mass frame can be expressed as

$$H\left(t\right)=T+H_{\text{hyp}}+H_{\text{ee}}+H_{\text{zee}}\left(t\right)$$ (1)

with the hyperfine contribution represented by

$$H_{\text{hyp}}=\xi_{A}{\vec{s}}^{\left(A\right)}{\vec{i}}^{\left(A\right)}+\xi_{B}{\vec{s}}^{\left(B\right)}{\vec{i}}^{\left(B\right)}$$ (2)

where $\xi_{i}$ denotes the hyperfine constant for the *i*th nucleus and ${\vec{s}}^{\left(i\right)}$ and ${\vec{i}}^{\left(i\right)}$ represent the spin and nuclear spin of the *i*th atom, respectively. In the case of two ^6^Li atoms, $\xi_{A}=\xi_{B}=\xi$ and ξ/h=152.1368407 MHz (*61*). T represents the usual kinetic energy operator in the basis of spherical harmonics. The electronic interaction between the outer electrons of two alkali atoms is described by the term

$$H_{\text{ee}}=V^{0}\left(r\right)P^{0}+V^{1}\left(r\right)P^{1}$$ (3)

where $V^{0}\left(r\right)$ and $V^{1}\left(r\right)$ are the singlet and triplet potentials while $P^{0}$ and $P^{1}$ are the corresponding projection operators (*62*, *63*). The dipole-dipole interaction for ^6^Li is very weak and therefore neglected in our calculations. We consider the time-dependent magnetic field

$$B\left(t\right)=B_{0}+B_{\text{rf}}^{\left(a\right)}\text{cos}2\pi \nu t+B_{\text{rf}}^{\left(b\right)}\text{cos}\left(4\pi \nu t+\phi \right)$$ (4)

where ν is the modulation frequency, $B_{\text{rf}}^{\left(a\right)}$ and $B_{\text{rf}}^{\left(b\right)}$ are the first and second harmonic amplitudes, and $B_{0}$ is the static bias field. This time-dependent magnetic field causes the Zeeman operator

$$\begin{array}{c} H_{\text{zee}}\left(t\right)=\mu_{B}[B_{0}+B_{\text{rf}}^{\left(a\right)}\text{cos}2\text{πν}t+B_{\text{rf}}^{\left(b\right)}\text{cos}\left(4\text{πν}t+\phi \right)\text{]}\\ \cdot \left[g_{e}s_{z}^{\left(A\right)}+g_{n}i_{z}^{\left(A\right)}+g_{e}s_{z}^{\left(B\right)}+g_{n}i_{z}^{\left(B\right)}\right] \end{array}$$ (5)

which accounts for the interaction between the magnetic field and the magnetic moment of the system to be time dependent. Where $\mu_{B}$ is the Bohr magneton, $g_{e}$ and $g_{n}$ are the electron and nuclear *g* factors, and $s_{z}^{\left(i\right)}$ and $i_{z}^{\left(i\right)}$ represent the *z* component of electron spin and nuclear spin for the *i*th atom, respectively.

Floquet theory states that for a time-periodic Hamiltonian H(t)=H(t+T), the solutions of the time-dependent Schrödinger equation can be written in the form $|\psi_{\beta }\left(t\right)\rangle =e^{-\left(i/\hbar \right)\epsilon_{\beta }t}|\phi_{\beta }\left(t\right)\rangle$, where $|\phi_{\beta }\left(t\right)\rangle$ are Floquet modes that are periodic in time with the same period T (*48*, *64*, *65*). The Floquet modes are solutions of the Floquet equation

$$\left[H\left(t\right)-i\hbar \frac{\text{∂}}{\text{∂}t}\right]|\phi_{\beta }\left(t\right)\rangle =\epsilon_{\beta }|\phi_{\beta }\left(t\right)\rangle$$ (6)

with quasienergies $\epsilon_{\beta }$.Because of the time periodicity, the Floquet modes and the Hamiltonian can be expanded into corresponding Fourier series $|\phi_{\beta }\left(t\right)\rangle =\sum_{n=-\infty }^{\infty }e^{-\text{in}\omega t}\left|\phi_{\beta }^{\left(n\right)}\right.\rangle$ and $H\left(t\right)=\sum_{n=-\infty }^{\infty }e^{-\text{in}\omega t}H^{\left(n\right)}$ with $\omega =\frac{2\pi }{T}$. Inserting these expansions into Eq. 6 and collecting terms of equal exponential factors leads to

$$\begin{array}{c} \sum_{n=-\infty }^{\infty }\sum_{l=-\infty }^{\infty }e^{-\text{in}\omega t}H^{\left(n-l\right)}\left|\phi_{\beta }^{\left(l\right)}\right.\rangle -\sum_{n=-\infty }^{\infty }\hbar \omega {\mathrm{ne}}^{-\text{in}\omega t}\left|\phi_{\beta }^{\left(n\right)}\right.\rangle =\epsilon_{\beta }\sum_{n=-\infty }^{\infty }e^{-\text{in}\omega t}\left|\phi_{\beta }^{\left(n\right)}\right.\rangle \end{array}$$ (7)

whereby the left term is derived by index shifting and making use of the infinity of the sums involved. Comparing the terms with coefficients $e^{-\text{in}\omega t}$, a set of coupled equations

$$\sum_{l=-\infty }^{\infty }\left[H^{\left(n-l\right)}-\delta_{n,l}\hbar \omega l\right]\left|\phi_{\beta }^{\left(l\right)}\right.\rangle =\epsilon_{\beta }\left|\phi_{\beta }^{\left(n\right)}\right.\rangle$$ (8)

can be derived. To recast this system into a time-independent matrix form suitable for numerical diagonalization, we define the extended state

$$\left|{\tilde{\Phi }}_{\beta }\;\right.\rangle =\sum_{n=-\infty }^{\infty }\left|\phi_{\beta }^{\left(-n\right)}\;\right.\rangle |n\rangle$$ (9)

where ∣n〉 denotes the Floquet index and introduce the operator

$$\begin{array}{c} \tilde{H}=H^{\left(0\right)}+\hbar \omega N+\sum_{k=1}^{\infty }\alpha^{k}H^{\left(k\right)}+\sum_{k=1}^{\infty }{\left(\alpha^{†}\right)}^{k}H^{\left(-k\right)} \end{array}$$ (10)

with $\alpha^{†}$ and α raising and lowering the Floquet index by one

$$\alpha^{†}|n\rangle =|n+1\rangle ,\alpha |n\rangle =|n-1\rangle$$ (11)

The number operator N is defined as

N∣n〉=n∣n (12)

With this, Eq. 8 can be seen to be an eigenvalue equation for the Floquet modes and quasienergies

$$\tilde{H}\left|{\tilde{\Phi }}_{\beta }\right.\rangle =\epsilon_{\beta }\left|{\tilde{\Phi }}_{\beta }\right.\rangle$$ (13)

It is instructive to examine the structure of $\tilde{H}$ closer, which reveals its equivalence to a quantized field Hamiltonian in the classical limit of large photon number. The first term $H^{\left(0\right)}$ contains all but the time-dependent part of the Zeeman term of Eq. 1. The second term corresponds to the energy of the rf field

$$H_{\text{rf}}=\hbar \omega N$$ (14)

Considering the time-dependent Zeeman term of Eq. 5 and restriction to ϕ=0 and ϕ=π, the last two terms of Eq. 10 give the atom-rf coupling

$$\begin{array}{c} H_{\text{cpl}}=\frac{\mu_{B}}{2}\left\{B_{\text{rf}}^{\left(a\right)}\left(\alpha +\alpha^{†}\right)+B_{\text{rf}}^{\left(b\right)}\left[\alpha^{2}+{\left(\alpha^{†}\right)}^{2}\right]\right\}\\ \cdot \left[g_{e}s_{z}^{\left(A\right)}+g_{n}^{\left(A\right)}i_{z}^{\left(A\right)}+g_{e}s_{z}^{\left(B\right)}+g_{n}^{\left(B\right)}i_{z}^{\left(B\right)}\right] \end{array}$$ (15)

with $B_{\text{rf}}^{\left(b\right)}$ being positive for ϕ=0 and negative for ϕ=π. With this, the total Floquet-Hamiltonian is given as

$$\begin{array}{c} \tilde{H}=T+H_{\text{hyp}}+H_{\text{zee}}^{\left(0\right)}+H_{\text{ee}}+H_{\text{rf}}+H_{\text{cpl}} \end{array}$$ (16)

where $H_{\text{zee}}^{\left(0\right)}$ captures the static part of Eq. 5. This time-independent Floquet-Hamiltonian is then expressed in the uncoupled and antisymmetrized basis (*63*)

$$\begin{array}{c} {\left[2\left(1+\delta_{\mathrm{msA},\mathrm{msB}}\delta_{\mathrm{miA},\mathrm{miB}}\right)\right]}^{-\frac{1}{2}}\\ \cdot \left[\left|L,M_{L};m_{\mathrm{sA}},m_{\mathrm{iA}};m_{\mathrm{sB}},m_{\mathrm{iB}},n\right.\rangle \right.\\ -{\left(-1\right)}^{L}\left.\left|L,M_{L};m_{\mathrm{sB}},m_{\mathrm{iB}};m_{\mathrm{sA}},m_{\mathrm{iA}},n\right.\rangle \right] \end{array}$$ (17)

before being diagonalized using the method of coupled-channel calculations, well suited for scattering problems. Floquet theory involves an infinite number of Floquet channels for a complete description of the system. However, in numerical calculations, the Floquet space must be truncated, and only a finite number of channels can be considered. If the basis of Eq. 17 is truncated to Floquet orders from $-\Delta_{\text{max}}$ to $+\Delta_{\text{max}}$, then a useful empirical guideline for selecting $\Delta_{\text{max}}$ is based on the ratio between the energies of modulation strength $\mu_{B}B_{\text{rf}}^{\left(a\right)}$ and the driving frequency hν

$$\Delta_{\text{max}}\geq 3\frac{\mu_{B}B_{\text{rf}}^{\left(a\right)}}{h\nu }$$ (18)

This criterion generally ensures sufficient convergence. However, it should be applied cautiously, and convergence must be verified manually for each specific case to ensure accuracy.

### Atom number measurement

The number of atoms is determined using absorption imaging on the $D_{2}$ line of ^6^Li. The cloud is imaged onto a sensitive scientific complementary metal-oxide-semiconductor (sCMOS) device with magnification M. From those images, the optical density

$$\text{OD}\left(x,y\right)=-\text{log}\frac{I\left(x,y\right)}{I_{0}\left(x,y\right)}$$ (19)

is determined where I is the intensity with and $I_{0}$ the intensity without atoms present. From this the number of atoms, N can be calculated by summing all pixels

$$N=\frac{A}{M\sigma_{0}}\sum_{\text{pixel}}\text{OD}\left(x,y\right)$$ (20)

where A is the pixel area size and $\sigma_{0}$ is the total absorption cross section. Fluctuations of the laser power, camera noise, and varying loading and transport efficiencies lead to fluctuating atom number measured for each experimental cycle. Limited knowledge of the absorption cross section leads to a systematic uncertainty in the total number of atoms present. Because we only need the relative number of atoms for our analysis, this issue is of no concern to us. By comparing the number of atoms with Floquet drive applied $N_{w}$ to the number of atoms remaining without the Floquet drive enabled $N_{w/o}$, we get the relative number of atoms $N_{\text{rel}}=\frac{N_{w}}{N_{w/o}}$.

### Coupled-channel calculations

The coupled-channel method solves the differential or equivalent integral equations for the radial coordinate, while the internal coordinates are diagonalized using a given basis set. Because of improved stability and ease of including the appropriate boundary conditions, we implemented a scattering code to solve the set of coupled Lippman-Schwinger integral equations using a spectral expansion into Chebyshev polynomials (*66*–*68*).

A requirement for accurate coupled-channel scattering calculations is the knowledge of very good molecular potentials. The potentials used for calculations of this work are based on published analytical potentials derived from spectroscopic data (*69*, *70*). These potentials were further improved by iteratively adjusting the potential, with the correction term described in (*71*), until the position of the I=2 Feshbach resonance matches the experimentally known value (*72*). For the numerical calculations of the AC Zeeman shift in Fig. 2 and two-color drive in Fig. 3, the potentials were additionally optimized to reproduce the measured resonance position for a modulation with frequency ν=13.1 MHz and strength $B_{\text{rf}}^{\left(a\right)}=8.9$ G at a collision energy of 724 nK.

The inelastic scattering cross section of Fig. 3C is a thermal average over the Boltzmann distribution $p\left(E_{c}\right)$ of collision energies $E_{c}$ (*73*). The thermally averaged inelastic cross section is then given as

$$\sigma_{\text{inel}}^{\text{avg}}=\int p\left(E_{c}\right)\sigma_{\text{inel}}\left(E_{c}\right)\;dE_{c}$$ (21)

where the cross section for a specific energy is calculated from the scattering length by (*74*)

$$\sigma_{\text{inel}}\left(E_{c}\right)=\frac{4\text{πIm}\left(a_{s}\right)}{k\left[1+k^{2}{|a_{s}|}^{2}+2k\text{Im}\left(a_{s}\right)\right]}$$ (22)

### Fitting the loss spectroscopic data

In case the additional slow modulation is used, the loss feature is symmetric and can be well captured using a sum of Gaussians

$$\begin{array}{c} N_{\text{rel}}\left(B\right)=N_{a}+\sum_{i}\frac{N_{i}}{\sigma_{i}\sqrt{2\pi }}\text{exp}\left[\frac{-{\left(B-B_{\text{res}}^{\left(i\right)}\right)}^{2}}{2\sigma_{i}^{2}}\right] \end{array}$$ (23)

to extract the resonance positions $B_{\text{res}}^{\left(i\right)}$, where $N_{a}$ is a constant offset accommodating for the background, $N_{i}$ scales the loss feature, and $\sigma_{i}$ gives its width.

If the modulation strength is sufficient and the precision of the magnetic offset field is good, then Floquet-Feshbach resonances can be studied without additional modulation to broaden the resonances. Then, the asymmetric nature of the losses can be seen. The lineshape of a Fano resonance

$$N_{\text{rel}}\left(B\right)=N_{1}\frac{{\left(q+\beta \right)}^{2}}{1+\beta^{2}}+N_{a}$$ (24)

with

$$\beta =\frac{B-B_{\text{res}}}{\Delta /2}$$ (25)

captures the observed loss profile to a high degree. The coefficient $N_{1}$ scales the resonance, and $N_{a}$ captures a constant offset. $B_{\text{res}}$ determines a resonance’s position, and Δ its width. The parameter q defines the shape of the resonance. For positive q, the loss minimum is toward the side of higher magnetic field values of a resonance, while for negative q, it is toward lower values. In the case q=0, the shape becomes symmetric.

### Analyzing the elastic response

To analyze the response of the atomic cloud to the trap quench, we took several measurements for each data point, stacked the resulting absorption images and fitted the cloud’s width with a Gaussian. The time evolution of the so extracted widths was fitted using

$$\sigma \left(t\right)=\sigma_{0}+{\mathrm{Ae}}^{-\gamma_{A}t}\text{cos}\left(\omega t+\theta \right)+{\mathrm{Be}}^{-\gamma_{B}t}$$ (26)

where $\sigma_{0}$ is the initial width and A and $\gamma_{A}$ the amplitude and decay rate of the oscillatory behavior with frequency ω and phase θ. The last term captures the cloud’s shrinkage due to nonoscillatory thermalization and trap losses with amplitude B and decay rate $\gamma_{B}$.

### rf circuit

**Fig. 5.**
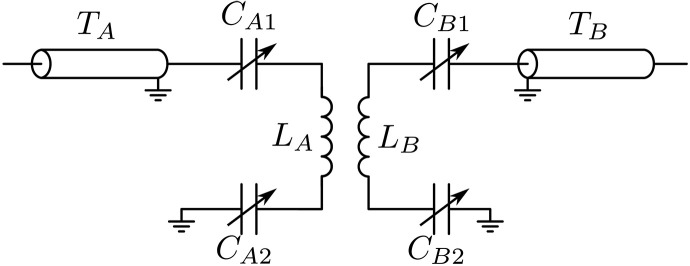
Schematic of the rf circuit. The circuit features two coupled, resonantly driven LC circuits, with variable capacitors for tuning the resonance frequency. Impedance matching to the 50-ohm amplifiers is achieved using quarter-wavelength transformers, constructed by connecting several coaxial cables in parallel.

The strong rf magnetic field modulation is generated using a pair of rf Helmholtz coils placed inside a set of high-current coils that produce the bias magnetic field $B_{0}$. These rf coils are part of two resonantly driven LC circuits, as shown schematically in Fig. 5. To match the low impedance of the LC circuits at resonance with the 50-ohm impedance of the amplifiers, we use quarter-wavelength transformers ($T_{A}$ and $T_{B}$ in Fig. 5) (*75*). These transformers are constructed by soldering several RG-58 50-ohm cables, cut to one quarter wavelength, in parallel. Fine-tuning of the resonance frequency is achieved through variable capacitors in each LC circuit. For single-frequency measurements, both LC circuits were tuned to the same frequency, whereas for the two-color drive, one circuit generated the first harmonic and the other the second harmonic.

### Calibrating the modulation strength

For good agreement between theory and experimental data, it is important to accurately measure the modulation strength produced by a given rf power at the place of the atoms. Fortunately, the atoms themselves provide a precise way to measure the magnetic field modulation. The rf dressing manipulates not only the scattering behavior of two atoms but also the energy levels of a single atom.

**Fig. 6.**
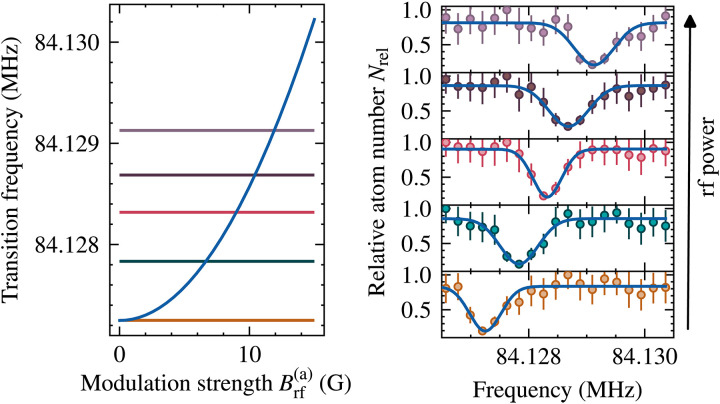
AC Zeeman shift of the ∣b〉→∣c〉 transition. The modulation shifts the transition frequency and hence allows for the accurate determination of the modulation strength with conventional methods of rf spectroscopy.

The standard method to precisely measure the static magnetic field $B_{0}$ is to drive spin flip transitions between hyperfine levels and infer the magnetic field strength from the observed transition frequency. The rf dressing leads to an AC Zeeman shift of the transition frequency and hence allows for the accurate measurement of the modulation strength, as demonstrated in Fig. 6.

In addition, pickup coils are placed in the stray fields. This provides a less accurate but still feasible way to measure the absolute modulation strength by comparing the induced voltage to numerical simulations to infer the magnetic field modulation strength at the atoms. From Lenz’s law, the magnetic field modulation at the pickup coil is found to be

$$B_{\text{rf}}=\frac{\sqrt{2}}{A2\pi \nu }\sqrt{\frac{P}{Z_{0}}}$$ (27)

where P is the measured rf power, $Z_{0}$ the characteristic impedance (typically $Z_{0}=50\;$ ohms), and A is the area enclosed by the pickup coil.
